# Non-thermal plasma-catalytic processes for CO_2_ conversion toward circular economy: fundamentals, current status, and future challenges

**DOI:** 10.1007/s11356-024-34751-3

**Published:** 2024-08-24

**Authors:** Ahmad Mukhtar, Sidra Saqib, Dinithi Mohotti, Robinson Jr. Ndeddy Aka, Mokter Hossain, Ekow Agyekum-Oduro, Sarah Wu

**Affiliations:** 1 Department of Chemical and Biological Engineering, University of Idaho, Moscow, ID 83843 USA; 2 Environmental Science Program, University of Idaho, Moscow, ID 83844 USA

**Keywords:** Circular economy, Heterogenous catalysis, Non-thermal plasma, Plasma catalysis, CO_2_ conversion, Energy efficiency

## Abstract

Practical and energy-efficient carbon dioxide (CO_2_) conversion to value-added and fuel-graded products and transitioning from fossil fuels are promising ways to cope with climate change and to enable the circular economy. The carbon circular economy aims to capture, utilize, and minimize CO_2_ emissions as much as possible. To cope with the thermodynamic stability and highly endothermic nature of CO_2_ conversion via conventional thermochemical process, the potential application of non-thermal plasma (NTP) with the catalyst, i.e., the hybrid plasma catalysis process to achieve the synergistic effects, in most cases, seems to promise alternatives under non-equilibrium conditions. This review focuses on the NTP fundamentals and comparison with conventional technologies. A critical review has been conducted on the CO_2_ reduction with water (H_2_O), methane (CH_4_) reduction with CO_2_ to syngas (CO + H_2_), CO_2_ dissociation to carbon monoxide (CO), CO_2_ hydrogenation, CO_2_ conversion to organic acids, and one-step CO_2_–CH_4_ reforming to the liquid chemicals. Finally, future challenges are discussed comprehensively, indicating that plasma catalysis has immense investigative areas.

## Plasma technology for CO_2_ conversion

Global climate change is considered an exponentially rising risk to the environment and human beings (Loenders et al. 2023, Zhang et al. 2022). It has been estimated that global warming has increased the earth’s temperature since the pre-industrial period by 1.5 °C, leading to irreversible changes and adverse effects on nature. Considering these alarming facts, it is necessary to put efforts into decreasing greenhouse gas emissions, mainly CO_2_ and CH_4_, which are reaching atmospheric concentrations of about 416 ppm and 1910 ppb, respectively (Cooley et al. 2022, Wang et al. 2023a). These figures indicate that CO_2_ and CH_4_ have increased by 19% and 173% since the pre-industrial era, respectively (Houghton et al. 2001). Besides, it is well known that society is much more dependent on fossil fuel combustion to gain energy, leading to considerable greenhouse gas emissions. Therefore, finding a potential solution for both problems is necessary, i.e., cutting greenhouse gas emissions and transitioning from fossil fuels to renewable and fuel-graded products, enabling the CO_2_ circular economy (Goeppert et al. 2014, Tcvetkov et al. 2019, Tebbiche et al. 2021). Although conventional thermal catalysis can fill the bill, the nature of the CO_2_ conversion reaction, i.e., highly endothermic (900–1273 K) for shifting the thermal equilibrium, makes it less energy efficient (Aramouni et al. 2018, Pakhare and Spivey 2014, Sun et al. 2023).

In thermochemical conversion processes, the higher thermal stability of the CO_2_ molecules demands much higher energy to break the double bonds in the CO_2_ molecule (O=C=O), making it less effective (Ashford and Tu 2017). Overcoming this issue by applying non-thermal plasma (NTP) is an innovative process to overcome the CO_2_ molecule activation challenges and convert it into various value-added and fuel-graded products. The reason is that the NTP can enable the reaction to proceed, which is thermodynamically unfavorable under ambient reaction conditions. Usually, the NTP performs at atmospheric pressure, low temperature, and non-equilibrium conditions and still achieves much higher conversion than the thermochemical conversion processes. Typically, the NTP produces energetic electrons having an average temperature (1 to 10 eV) possessing a high capability of CO_2_ molecule activation by ionization, followed by excitation, and finally, dissociation, leading to the development of an avalanche of reactive species, which include the ions, excited atoms, radicals, and molecules which can trigger the chemical reaction into forward direction (Bogaerts and Neyts 2018, Mehta et al. 2019, Tu and Whitehead 2012, Tu et al. 2019, Zeng et al. 2018). The significant benefit of NTP technology is the ease of installation, compactness, flexibility, and the considerable potential of integration with other technologies for effective and energy-efficient conversion of CO_2_ to value-added and fuel-graded products (George et al. 2021).

The major obstacle in the pathway of the NTP technology is the improvement of energy efficiency, which could be accomplished by combining the catalyst and transforming the plasma process to the hybrid plasma catalysis process, creating synergy, and providing enough room for the excited species for catalyst surface interaction and subsequently lose their excitation energies and relax before reaction. However, the knowledge available for the plasma catalysis process to effectively and efficiently convert CO_2_ to value-added and fuel-graded products and, most importantly, for the complex interactions between plasma, catalyst, and reactive species is minimal (Fridman 2008).

This review paper comprehensively focuses on plasma and plasma catalysis technologies, their fundamental physicochemical properties; integration in reactors with different configurations; and possible interactions between complex plasma, catalyst, and reactive species. In addition, the general mechanism of the plasma-only process for CO_2_ activation by ionization, excitation, and dissociation is also discussed, along with current developments and future challenges in plasma catalysis for different CO_2_ conversion routes.

## Comparison of plasma with conventional CO_2_ conversion approaches

**Fig. 1:**
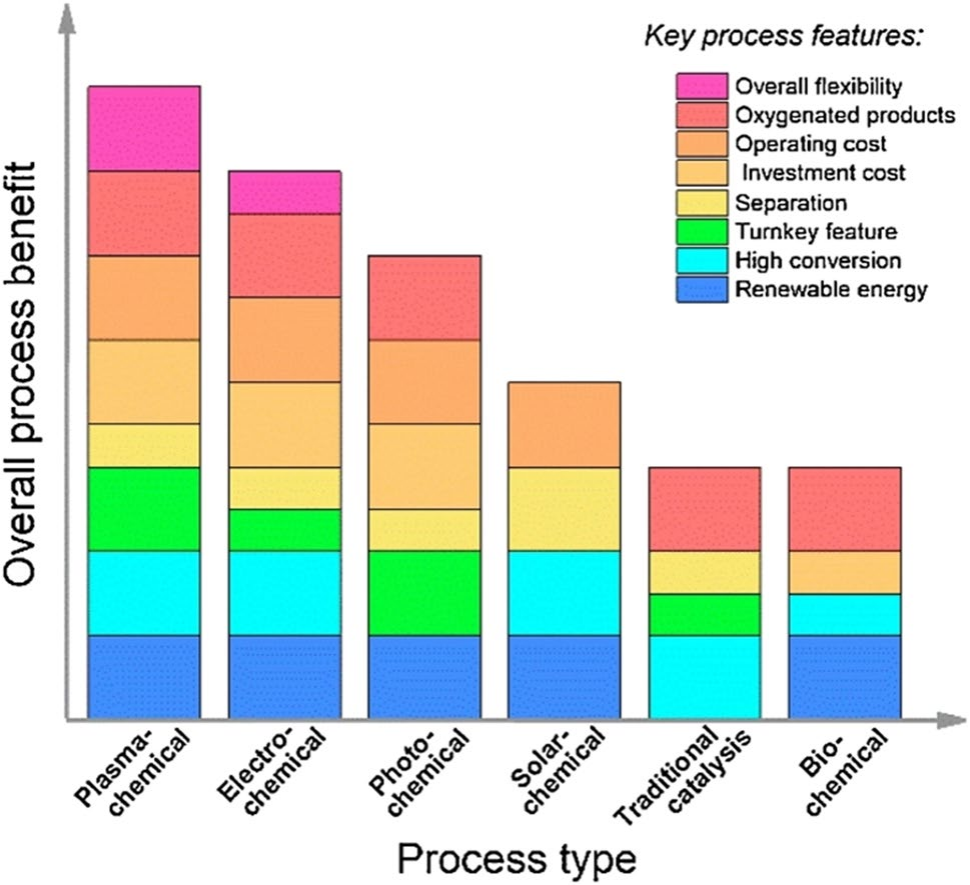
Comparison of overall advantages of traditional CO_2_ conversion technologies with plasma-catalysis. Reprinted from the references (Chen et al. 2021, Snoeckx and Bogaerts 2017) with the permission of Elsevier and the Royal Society of Chemistry

Comparison has been summarized in Table 1 for all traditional and conventional CO_2_ conversion technologies and the plasma-only and plasma-catalysis technologies. Overall, it has been found that the conventional thermochemical conversion of CO_2_ assisted by catalysts cannot split CO_2_; however, it can be effectively implemented to dry reforming methane (DRM) and the hydrogenation of CO_2_. At the same time, data for CO_2_ reduction in H_2_O is not available for the catalyst-assisted thermochemical conversion approach. Compared to the catalyst-assisted thermochemical conversion approach, all other novel approaches successfully achieved CO_2_ reduction in H_2_O, and the electrochemical and solar-assisted thermochemical conversion approaches can also successfully split the CO_2_. Interestingly, the literature generally does not report the CH_4_ and hydrogen (H_2_) combination. Although plasma-chemical technology is the only technology discussed here that can be successfully applied to all four significant areas of CO_2_ conversion research, including DRM, CO_2_ hydrogenation, CO_2_ splitting, and CO_2_ reduction in H_2_O, CO_2_ reduction in H_2_O is not investigated in the plasma-catalysis system but only in the plasma-only system. In all four CO_2_ conversion routes, DRM and CO_2_ splitting are considered the two most important routes and have already been demonstrated to achieve 90–95% and 60% energy efficiency with the plasma-only treatment, respectively. Even though the one-step synthesis of value-added liquid products from CO_2_ conversion in the plasma-catalysis mode has been proved by preliminary research, more research is still needed to improve the process. CO_2_ reduction with H_2_O, also known as the “artificial photosynthesis,” is the least mature technology and requires significant research to improve. It is admitted that a clear priority should be given to plasma catalysis owing to its several key benefits, including flexibility, versatility, and low operation cost, as compared in Fig. 1.

**Table 1:**
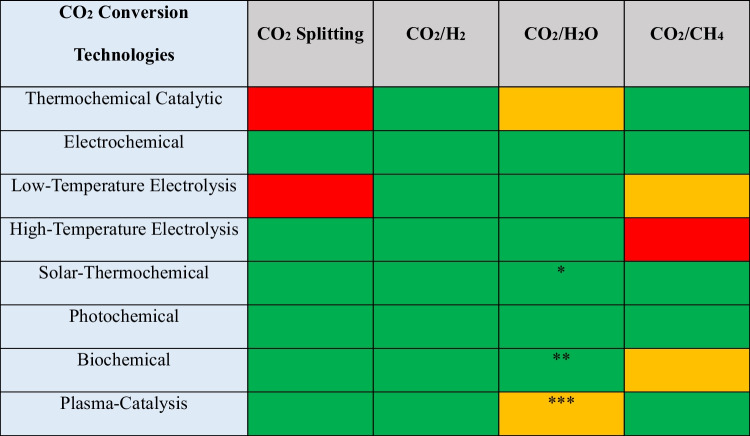
A schematic comparison of different technologies for CO_2_ conversion in different routes (Hecimovic et al. 2024, Snoeckx and Bogaerts 2017)

Green: efficient, yellow: to be proven, and red: inefficient

*CO_2_ and H_2_ are not converted simultaneously

**H_2_O is the vital nutrient for the algae growth

***When used hybrid plasma-catalysis mode

## Different types of plasmas

The CO_2_ conversion process employs various types of plasma, as described in this section: dielectric barrier discharge, radiofrequency inductively coupled plasma, atmospheric pressure glow discharges, gliding arc plasma, corona discharge, and microwave plasma. A table detailing their operational characteristics is provided in the following section (Table 2).

**Table 2:** Operating characteristics of different types of plasmas used in the CO_2_ conversion processes (Chen et al. 2021, Ong et al. 2022)

Plasma types	Operating pressure (mbar)	Electron density (cm^−3^)	Electron temperature (*T*_*e*_, eV)	Gas temperature (*T*_*g*_, K)	References
DBD	atm	10^12^–10^15^	1–30	300–500	Bogaerts and Centi (2020), Bogaerts et al. (2015), Kutz (2011), Okubo et al. (2018), Puliyalil et al. (2018), Snoeckx and Bogaerts (2017), Yamasaki et al. (2020)
GAC	atm	10^11^–10^15^	1.4–2.1	1000–3000	Bogaerts and Snoeckx (2019), Li et al. (2020), Nunnally et al. (2011), Puliyalil et al. (2018), Ramakers et al. (2017), Snoeckx and Bogaerts (2017), Trenchev andBogaerts (2020), Zhang et al. (2014), Zhu et al. (2020)
APGD	10^−5^–atm	10^9^–10^12^	1–2	~ 2000	Arumugam et al. (2018), Ghorbanzadeh et al. (2009), Kiruthika andShanmugavelayutham (2020), Kutz 2011, Li et al. (2019), Tao et al. 2011, Wissel et al. (2013)
CD	atm	10^8^–10^14^	3.5–5	< 400	Butterworth et al. (2016), Fridman (2008), Kutz (2011), Michielsen et al. (2017), Mikoviny et al. (2003), Nguyen et al. (2015), Schutze et al. (1998), Tao et al. 2011, Xu et al. (2004), Yang (2002)
RCP	10^−3^–atm	10^12^–10^15^	0.65–1.85	~ 400^a^	Fridman (2008), Hopwood (1992), Kwak et al. (2015), Nguyen (2009), Okumura (2010), Park et al. (2001), Spencer andGallimore (2011), Wang et al. (2017a), Winchester and Payling (2004)
MW	10^−5^–atm	10^10^–10^15^	0.4–0.9	2000–6000	Azizov et al. (1983), Bongers et al. (2017), Britun et al. (2017), Chen et al. (2015), Fridman (2008), Hong et al. (2006), Hrycak et al. (2014), Jasiński et al. (2013), Legasov et al. (1978), Mizeraczyk et al. (2014), Snoeckx and Bogaerts (2017), Tao et al. (2011), van den Bekerom et al. (2019)

*DBD* dielectric barrier discharge, *GAC* gliding arc plasma, *APGD* atmospheric pressure glow discharges, *CD* corona discharge, *RCP* radiofrequency inductively coupled plasma, *MW* microwave plasma

^a^At an input power < 35 W

Dielectric barrier discharge (DBD) can be formed by employing the electrical field linking two electrodes as a non-uniform discharge with an alternating (AC) current with at least one electrode covered with a dielectric barrier. Different arrangements of the two electrodes could be made to minimize the gas bypassing the plasma area by keeping both electrodes parallel. However, a cylindrical configuration is more appropriate for greenhouse gas conversion applications. The advantage of DBD is that it can operate near ambient pressure and temperature. Due to its simple design, it can be easily upgraded for commercialization. In the case of DBD, the conversion efficiency of CO_2_ is appropriate; however, the energy efficiency is 2–10% less than that of other plasmas (Bogaerts and Centi 2020, Okubo et al. 2018, Ong et al. 2022, Puliyalil et al. 2018, Yamasaki et al. 2020).

Gliding arc plasma can work at ambient pressure and be produced by applying electricity between two electrodes with flat diverging. Initially, an arc is formed at the inter-electrode with the shortest distance. After that, the arc glides along with the surfaces of the diverging electrode, leading from the small to longer distances between the electrodes in the direction of gas flow until it extinguishes. The process of arc formation is then repeatedly reformed immediately at the initial sport. It has comparatively better energy efficiency even at ambient pressure with a range of 43–60% splitting CO_2_ and DRM conversion of 18% and 8–16%, respectively. The short residence time of the gas to be treated is a significant constraint of the traditional gliding arc plasma; however, the residence time can be increased with modifications in design (Bogaerts and Snoeckx 2019, Nunnally et al. 2011, Puliyalil et al. 2018, Ramakers et al. 2017).

As the name implies, atmospheric pressure glow discharge is operatable at ambient pressure with advantages over other plasmas, such as higher electron density and proper plasma temperature. The plasma of the discharge is luminous or glowing. It is formed by employing voltage ranging between a few hundred and a few kilovolts between two electrodes with a ballast circuit to minimize the transition of glow to arc. It has been reported that by using atmospheric pressure glow discharges with an input power of 23 W, the CH_4_ and CO_2_ conversion of 61% and 50% can be achieved, respectively (Arumugam et al. 2018, Ghorbanzadeh et al. 2009, Li et al. 2019, Tao et al. 2011).

Corona discharge is a non-uniform discharge with a lower current density that can be produced at ambient pressure. It usually involves two different electrodes, including a plate with a low curvature and a sharp tip with a high curvature. In such arrangements, when high voltage is implemented between such two different electrodes, an electric breakdown near the sharp tip with high curvature leads to plasma formation in the drift region. In the reported work, it has been demonstrated that by using the corona discharge with 46.3 W of discharge power, the conversion of CH_4_ and CO_2_ was 62.4% and 47.8%, respectively (Nguyen et al. 2015, Schutze et al. 1998, Tao et al. 2011, Yang 2002).

Radiofrequency inductively coupled plasma can be formed into the plasma chamber by employing the electromagnetic field or, more precisely, radio frequency field, and the radio frequency field can be produced using the radio frequency power through the dielectric window to the planar coil. Compared to other types of plasmas, the radiofrequency inductively coupled plasma has less than 50% energy efficiency, which could be significantly decreased at higher power, e.g., > 100 kW (Hopwood 1992, Kwak et al. 2015, Okumura 2010, Wang et al. 2017a).

Microwave plasma can be formed by implementing microwave power to a gas-filled quartz tube, and the gas temperature can be easily raised to > 3000 K at (sub)ambient pressure with energy efficiency up to 40% comparatively higher at atmospheric pressure and normal flow conditions. However, this energy efficiency can be increased to 90% with a CO_2_ conversion between 10 and 20% under specific operational conditions such as the supersonic/subsonic gas flow and reduced pressure. The catalyst cannot be investigated quickly for the higher gas temperature in microwave plasma. Still, it can be placed after the plasma reactor, also known as the “post-plasma catalysis,” although only a few studies have been reported (Azizov et al. 1983, Fridman 2008, Hong et al. 2006, Hrycak et al. 2014, Jasiński et al. 2013, Legasov et al. 1978, Mizeraczyk et al. 2014, Tao et al. 2011).

## Plasma-catalysis reactor configurations

**Fig. 2:**
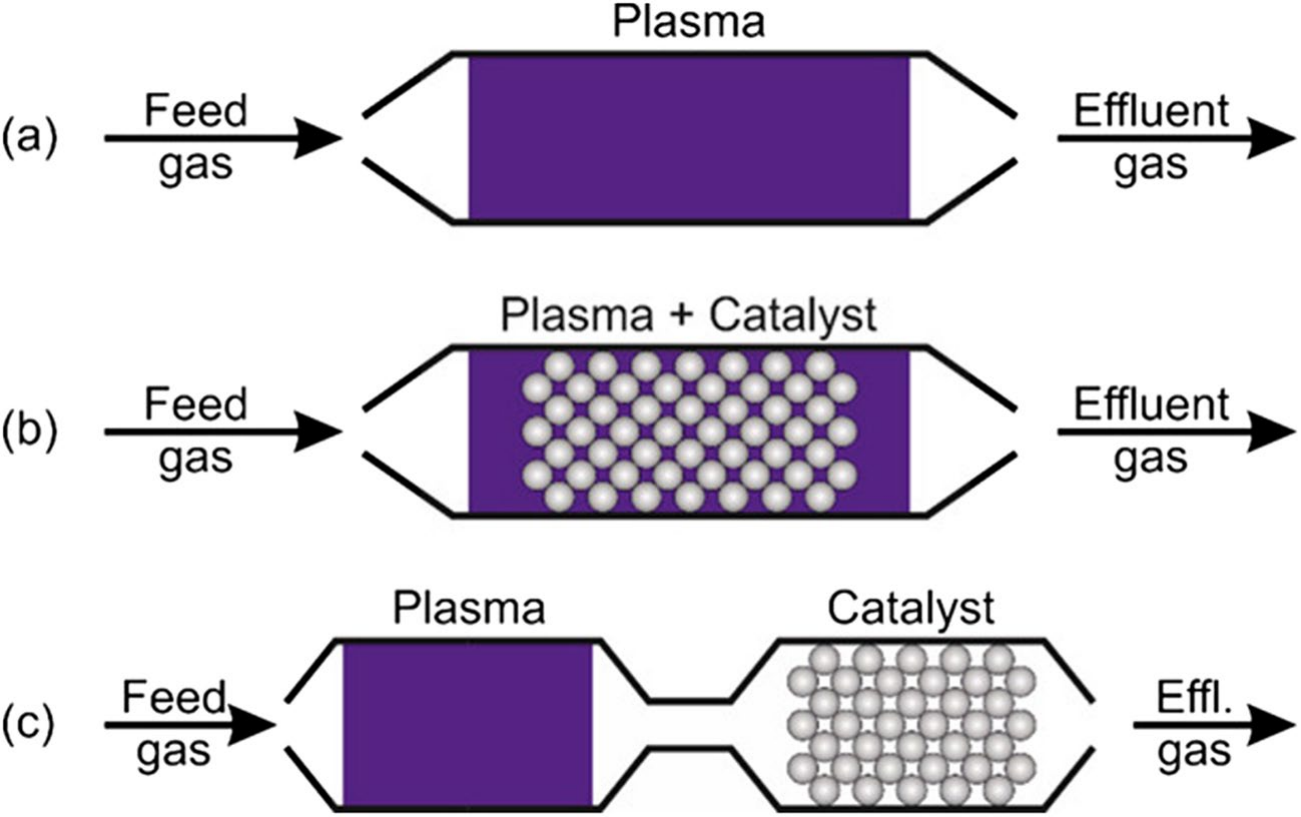
Schematic illustrations of the reactor configurations for **a** plasma-only, **b** IPC, and **c** PPC. Reprinted from the reference (Ollegott et al. 2020) with the permission of Wiley

It is well known that a series of parameters are involved in the plasma-only process (Fig. 2) for effective and energy-efficient conversion of CO_2_ to the value-added products, including the specific energy input (SEI), composition of the feed gas, feed gas flow rate, discharge power, dielectric material, electrode configuration, discharge length/gap, and discharge frequency and type (George et al. 2021). However, coupling the plasma with the catalyst provides cooperative effects as the plasma-produced species can interact at the surface of the catalyst due to the interactive behavior of plasma and catalyst (Tu et al. 2011, Tu and Whitehead 2012, Whitehead 2016, 2019). There are two significant configurations in plasma catalysis, known as the in-plasma and the post-plasma catalysis, represented as IPC and PPC, respectively, as shown in Fig. 2.

The IPC-configured reactor (Fig. 2) is based on a single-step procedure where a catalyst is placed in the plasma discharge region to enable to produce the short- and long-lived reactive species by the NTP to immediately interact with the surface of the catalyst with the plasma zone, leading to enhancement of CO_2_ conversion and the targeted product selectivities. The IPC-configured reactor facilitates energy efficiency as the required energy to produce the short-lived species is often wasted either in the plasma-only or the PPC-configured reactor. In the IPC-configured reactor, the catalyst is either partially or fully packed into the plasma discharge region, and such packing configuration also affects the discharge properties. Due to the potential impacts of plasma on the catalyst, as discussed in the preceding sections, the NTP can synthesize and modify the catalyst to improve its performance and stability, a key driver towards commercialization (Di et al. 2019, Tu et al. 2013, Wang et al. 2018b, Witvrouwen et al. 2012).

The PPC-configured reactor (Fig. 2) facilitates the two-stage reactions where the gas-phase reaction occurs in the plasma region, followed by the second stage at the catalyst’s surface. In this case, as the catalyst is not within the plasma discharge region, the catalyst bed is heated thermally to promote the reaction at the catalyst’s surface. One of the disadvantages of the PPC-configured reactor is that as it is placed outside the plasma discharge region, only long-lived species and end-products can travel from the plasma region to the catalyst surface to interact with the catalyst surface for the reaction (Bo et al. 2020, Neyts et al. 2015). The short-lived species are the vibrationally excited species with 1–10 nanoseconds of short lifetimes at atmospheric pressure and seem difficult to survive while transitioning from the plasma region to the catalyst region. Hence, the PPC-configured reactor appears not feasible for efficient CO_2_ conversion due to the lack of short-lived species conversion to targeted products.

## Current developments in NTP-assisted CO_2_ conversion

### CO_2_ reduction with 0H_2_O

Chen et al. (2015) reported the concurrent CO_2_ and H_2_ dissociation using a surface wave sustained discharge in a pulse regime to investigate the potential impact of various process parameters, comprising the CO_2_/H_2_O feed flow rate ratio and SEI on the energy efficiency and conversion by identifying and quantifying the product streams using gas chromatography. It has been found that the H_2_ and carbon monoxide (CO) formation in the same gas mixture can be carried out at the optimum SEI value of 1.6 eV/molecule. Further, this process is favored by the low feed flow rates and maximum energy efficiency. It has been found that the lower SEI and CO_2_ with an excess amount behave as a “physical catalyst” for H_2_O, leading to the formation of H_2_.

In contrast, the higher SEI and feed flow rates significantly reduced the H_2_ yield, suggesting that the CO formation consumes H_2_, thus facilitating the reverse direction of WGSR. The optimal emission spectroscopy (OES) results revealed that the lower gas temperature favored the higher dissociation rate. Hoeben et al. (2015) investigated that the CO_2_ conversion with H_2_O to produce CH_4_ was observed using a pulsed corona discharge at a higher density using a CO_2_ flow over the H_2_O film, which results in the formation of CH_4_ under mild reaction conditions. It was interesting to know that the CO_2_ and H_2_O dissociation using plasma induces the CO_2_ or CO hydrogenation chemistry over the NiCr wire materials, which behave as catalysts superior to low-alloy steel.

### CH_4_ reforming with CO_2_ to syngas

Nearly two decades ago, Huang et al. (2000) reported the reformation of CO_2_–CH_4_ by a glow discharge plasma using and without using micro-arc formation with a Y-type reactor. The process seems effective for converting the mixture of CO_2_ and CH_4_ into syngas (CO and H_2_) as a dominant product with a minor amount of hydrocarbons also identified. Interactions between the different species and arms were observed even when only reactants were excited. With micro-arc formation, the CO selectivity was enhanced, and the process became more energy efficient than the one without micro-arc formation. However, they found that the conventional catalytic methods have better energy efficiency than the plasma-only process to convert CO_2_ and CH_4_ mixture to produce syngas. In another similar work (Li et al. 2002), the product distribution generated from the CO_2_ and CH_4_ mixture was investigated with the impact of the discharge gap width, feed gas composition, and post-glow zone effects. From the feed gas composition effect, the gaseous and liquid hydrocarbons increased with an enhancement in the feed concentration of CH_4_. At the same time, the CO selectivity was found to be directly proportional to the feed concentration of CO_2_. The CO_2_–CH_4_ conversion is enhanced at a shorter discharge gap, favoring the synthesis of liquid-phase hydrocarbons and organic acids.

For this reason, an atomic economic reaction was identified to produce acetic acid directly from CO_2_–CH_4_. In addition, the higher discharge gap resulted in the synthesis of methanol (CH_3_OH) and ethanol (C_2_H_5_OH). Most liquid-phase hydrocarbons identified in this work were highly chained, with olefins about 5.5 wt.%.

For the first time, Patiño et al. (2005a) investigated the application of radio frequency in the plasma-based to convert the CH_4_ and CO_2_ mixture to syngas with a conversion achieved similar to the other processes, which was further enhanced by increasing the radio frequency power and feed gas pressure. The reforming of CH_4_ was investigated by injecting steam, CO_2_, and oxygen (O_2_) separately for reaction with CH_4_, and it was found that the steam plasma was the most effective. In contrast, the O_2_ plasma was the most oxidant. Higher power and higher H_2_O–CH_4_ feed flow rates resulted in CO_2_-free syngas. In contrast, lower feed flow rates at the power of 50 W produced CO_2_–CO-free H_2_, indicating their potential for clean H_2_ production for fuel cell applications. This way, an H_2_ to CO ratio of up to 16 could be produced at certain reaction conditions.

Qi et al. (2006) found that it could be a potential option to produce syngas from CO_2_ and CH_4_ reforming using an abnormal glow plasma due to its unique energy density and the temperature profile in the discharge space. With an enhancement in either the energy density with temperature in the discharge space or power, the CO_2_ and CH_4_ conversion increased without any potential impact on the CO and H_2_ selectivity, possibly because the electron’s energy distribution did not change during the discharges. A competing reaction between the oxygen, carbon, molecules, and hydrogen radical atoms could increase the H_2_ to CO ratio, which can be achieved by increasing the CH_4_/CO_2_ feed ratio. However, this strategy could lead to carbon deposition on electrodes, which can be avoided by adding oxygen to the reaction system.

Goujard et al. (2009) investigated the synthesis of hydrocarbons and syngas from biogas (excessive CH_4_, CH_4_/CO_2_ = 1.5) using NTP, where the CH_4_ and CO_2_ conversion depended on the discharge power despite the variation in frequency or voltage at room temperature. In contrast, the excessive CO_2_ was favorable for higher CH_4_ and higher conversion of CO_2_, leading to higher CO selectivity. Conversely, the higher CH_4_ concentration favored hydrocarbon synthesis, and higher CO_2_ concentration increased the selectivity towards CO. The results showed that temperature significantly influenced selectivity with higher CO selectivity at elevated temperatures, i.e., 873 K. These results could be elaborated by considering the reaction between the active oxygen species and the carbon formed from the CH_4_ cracking. In this regard, the catalyst facilitates CO_2_ activation and favors the higher CO selectivity in a hybrid plasma system compared to a plasma-only system at 773 K. More detailed, the catalyst’s metallic nickel (Ni) species behaved as a radical trap. At the same time, the La_2_O_3_-based basic sites activated the CO_2_ immediately as the catalyst was incorporated with the discharge plasma.

Tu and Whitehead (2014) reported a hybrid plasma-catalysis process to produce clean fuel and value-added products by reforming the CO_2_–CH_4_ mixture using an AC gliding arc reactor. It has been found that using the DBD reactor for CO_2_–CH_4_ reforming produced a wide range of hydrocarbons, while using the AC gliding arc plasma resulted in much cleaner fuel, and of course, syngas was the dominant product. It could be attributed to the high electron density generated by AC gliding arc plasma, which may cause a change in the reaction pathway. But this postulate needs further investigation. The optical emission spectra identified various species, including Al, CH, C_2_, O, CO, H_*α*_, and H_*β*_. Compared to the hydrocarbons and syngas, a series of varying carbon nanomaterials were also formed, which included the spherical carbon nanoparticles, amorphous carbon, and multi-walled carbon nanotubes, indicating that this hybrid process could open new doors to synthesizing carbon nanomaterials in more sustainable and energy-efficient ways.

**Fig. 3:**
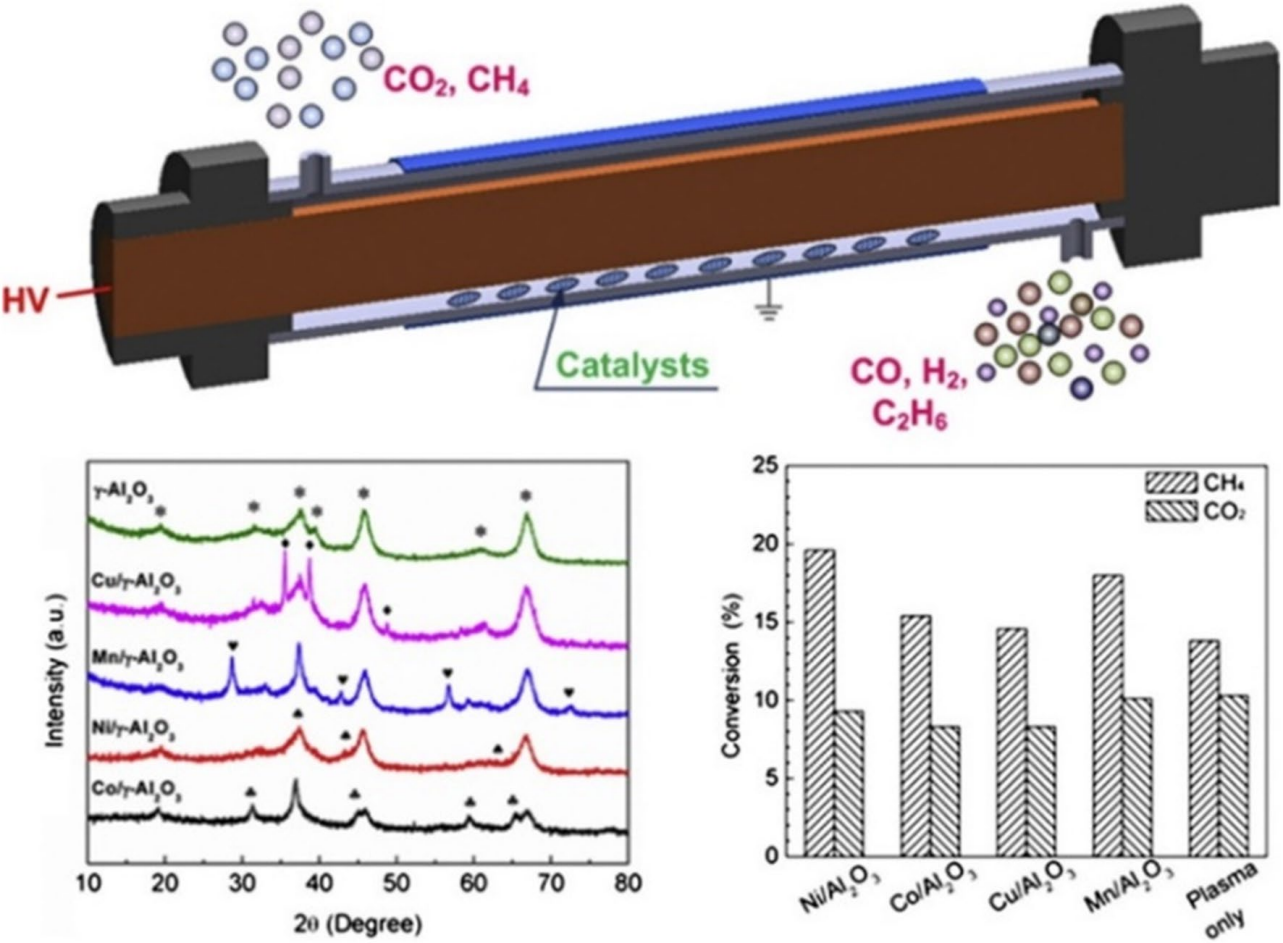
Effect of catalysts and textural properties on the CO_2_*–*CH_4_ reforming to syngas and other value-added products in plasma-only and plasma-catalysis systems. Reprinted from the reference (Zeng et al. 2015) with the permission of Elsevier

In another work, Zeng et al. (2015) reported the potential impacts of the hybrid plasma catalysis system on the CO_2_–CH_4_ reforming using the alumina-supported metal (Co, Mn, Cu, and Ni) catalysts using a coaxial DBD reactor. It has been observed that with the plasma-only mode, the CO_2_–CH_4_ conversion to syngas was strongly influenced by the feed ratio, not the feed flow rate; however, combining the Ni and Mn supported on alumina catalyst with plasma by packing into the discharge gap demonstrated a synergistic effect on the conversion of CH_4_–CO_2_ to syngas. Still, the CO_2_ conversion was not affected by the catalyst presence. The conversion was almost independent of the textural characteristics of the employed catalyst (Fig. 3).

Snoeckx et al. (2015) reported the CH_4_–CO_2_ reforming to syngas using the DBD plasma to identify the most promising parameter among the residence time, power, frequency, and gas mixture composition to determine the most effective and energy-efficient condition from the commercialization point of view. They optimized the abovementioned parameters and obtained the energy efficiency and maximal conversion, which were found to be 8.5% and 84%, respectively. In general, they found that the higher concentration of CO_2_ favored energy efficiency and higher conversion; however, with an enhancement in the SEI, which accounted for both residence time and power, the results revealed only an enhancement of the conversion with a slight decline in the energy efficiency. Among the different parameters investigated, the most complicated effect came from the frequency. The product of the residence time and frequency, which indicated the total counts of filaments undergone by the gas molecules while passing via the reactor, was a decisive factor. It has been found that the higher counts of micro-discharge filaments with less energy per filament resulted in higher energy efficiency and conversion.

Another similar work was reported by Nguyen et al. (2015) for reforming CO_2_–CH_4_ to syngas using the corona discharge plasma with CO and H_2_ as dominant products. Among different process parameters, such as the total feed flow rate, applied peak voltage, CO_2_/CH_4_ ratio, and pulse frequency, the pulse frequency and CO_2_/CH_4_ ratio were the most significant parameters. Overall, it has been found that a decrease in the total feed flow rate with an enhancement in the pulse frequency and peak voltage resulted in a higher conversion of CO_2_/CH_4_. In addition, the CO selectivity decreased more than that of H_2_ with the increased CO_2_/CH_4_ ratio. With a rise in the applied peak voltage up to 10 kV, the CO and H_2_ selectivity increased and then decreased upon further increasing the applied peak voltage. In contrast, the CO and H_2_ selectivity was not influenced by the variation in the pulse frequency or total feed flow rate.

Scapinello et al. (2016) reported CO_2_–CH_4_ reforming to syngas using a pulsed (NRP) discharge plasma (nanosecond repetitively) and reached a series of observations and conclusions. They found that the utilization of NRP discharge plasma demonstrated the highest performance, with an increased CH_4_ and CO_2_ conversion as a function of SEI. Due to the competing production of carbon powder and water, the selectivity towards syngas was decreased. To improve energy efficiency, water formation should be reduced with a thorough understanding of the water formation mechanism in this process.

**Fig. 4:**
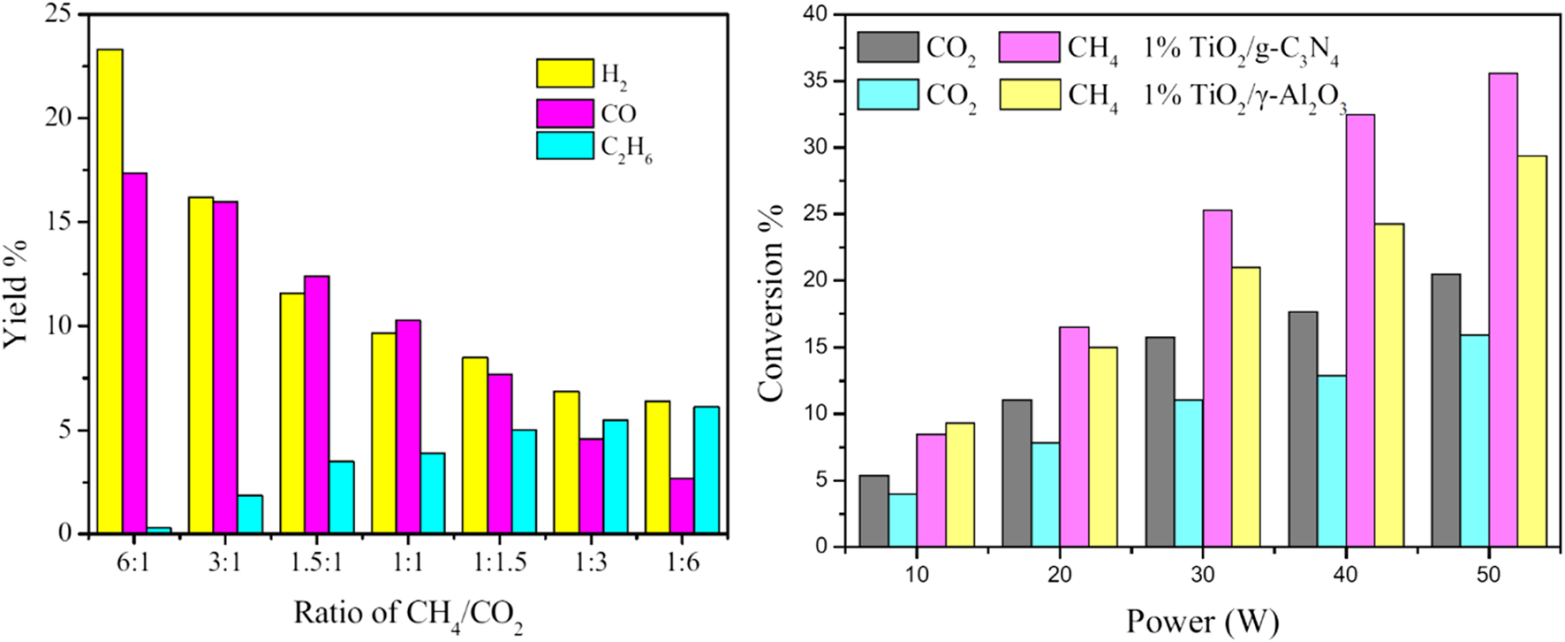
(Left): effect of the CH_4_/CO_2_ ratio on the syngas and other product yields in the plasma-only system. (Right): effect of power and catalyst on the CH_4_/CO_2_ reforming conversion in the plasma-catalysis system. Reprinted from the reference (Lu et al. 2017) with the permission of Springer

Lu et al. (2017) reported CO_2_–CH_4_ reforming to syngas with a cooperative effect of the plasma with a catalyst supported on the graphitic carbon nitride (g-C_3_N_4_) using a DBD reactor. In the case of the plasma-only system, the overall reaction was affected by the overall feed flow rate, CH_4_/CO_2_ ratio, and input power, indicating an increased conversion of CO_2_ and CH_4_ with a rise in the input power and a decline in the total feed flow rate. However, the syngas selectivity was probably reduced due to coke formation at elevated energy levels. In the case of a hybrid plasma-catalysis system, the conversion of CO_2_ and CH_4_, as well as the syngas yields, was higher because of the synergistic effect of the plasma and the catalyst (Fig. 4). By increasing the mass ratio of TiO_2_, the reaction probability between the catalyst and the higher energy electrons was reduced due to the adsorption of higher energy electrons.

Yap et al. (2018) investigated CO_2_–CH_4_ reforming to syngas under NTP, with the catalyst (La_2_O_3_/Al_2_O_3_) filling the entire zone of discharge in helium gas. It has been observed that the presence of helium in the feed gas favored the higher CO_2_/CH_4_ conversion, probably due to the transfer of energy from the activated helium species to the reactant molecules and decreasing the carbon deposition. Overall, helium utilization was not found to be favorable in terms of energy efficiency.

**Fig. 5:**
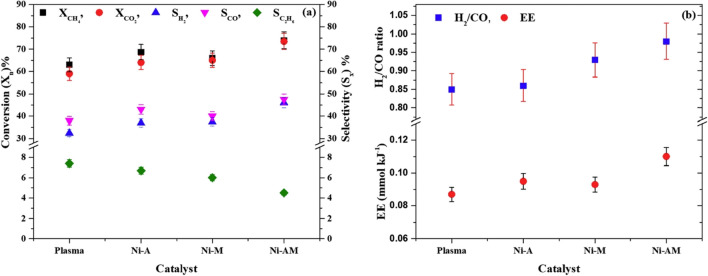
Effect of the different catalysts on the (left) CH_4_/CO_2_ reforming conversion (X) and selectivity (S), (right) H_2_/CO ratio, and energy efficiency (EE). Reprinted from the reference (Khoja et al. 2018) with the permission of Elsevier

Khoja et al. (2018) investigated a Ni/γ-Al_2_O_3_-MgO catalyst in the packed-bed DBD plasma reactor for CO_2_–CH_4_ reforming to syngas and additional value-added products. Performance was compared with the plasma-only system. It has been found that Ni/γ-Al_2_O_3_-MgO resulted in low-carbon deposition compared to Ni/MgO and Ni/γ-Al_2_O_3_, probably because of the higher metal dispersion, high Lewis’s basicity, and surface faceting (Fig. 5). The lowest carbon deposit was observed with the equimolar formation of CO and H_2_. This lower carbon formation was due to suppressing the carbon gasification and reversing the water gas shift reaction (RWGS). In addition, the higher H_2_ production was credited with suppressing the recombination of H and CH_3_ species to form CH_4_.

**Fig. 6:**
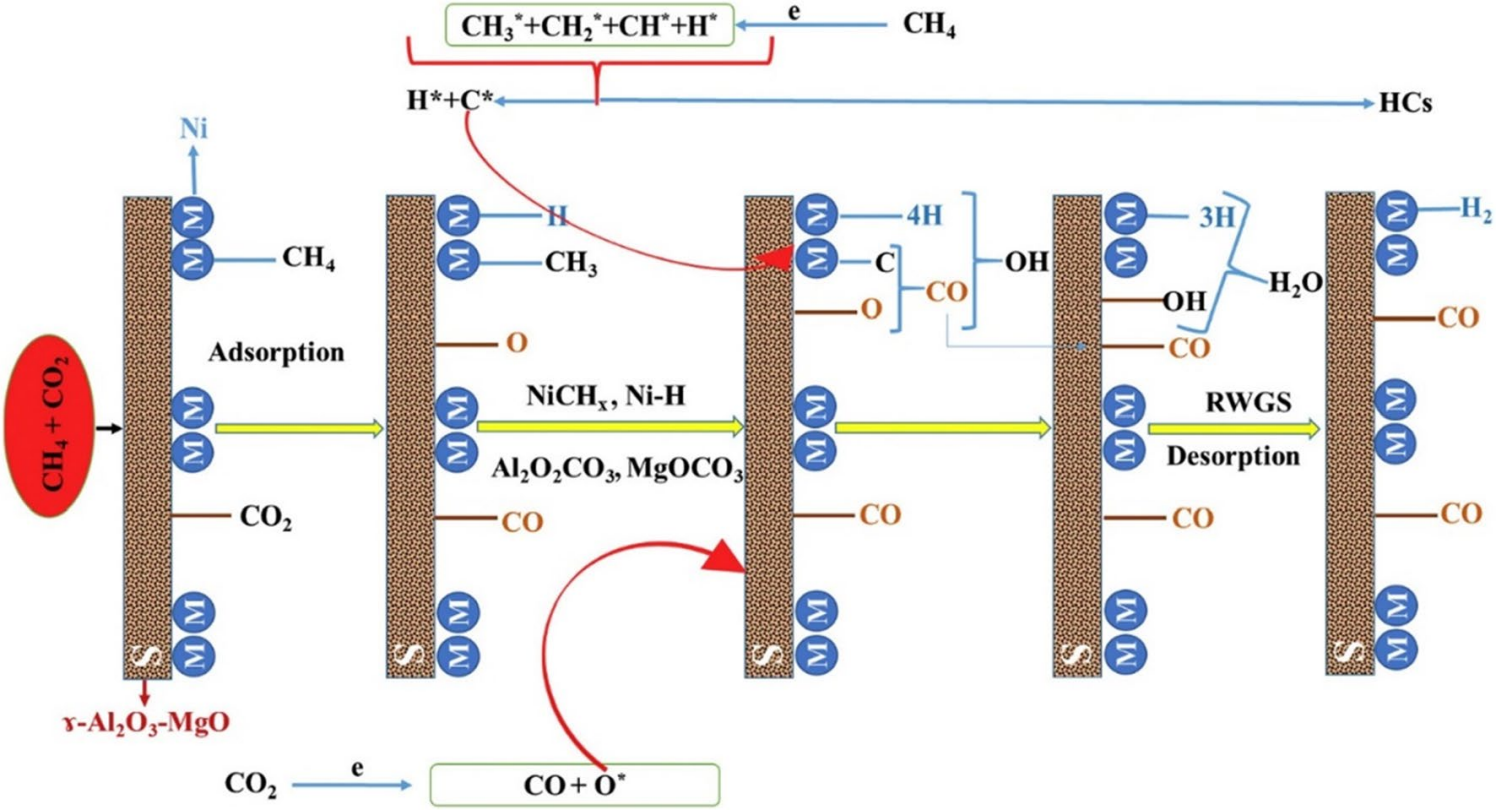
A proposed reaction mechanism for the CO_2_*–*CH_4_ reforming over the Ni/γ-Al_2_O_3_-MgO catalyst in the presence of plasma. Reprinted from the reference Khoja et al. (2018) with the permission of Elsevier

A detailed reaction mechanistic pathway was proposed for the hybrid plasma-catalyst system to produce syngas and other value-added products through CO_2_–CH_4_ reforming. The proposed mechanism was believed to follow the Langmuir-Hinshelwood-Hougen-Watson (LHHW) mechanism with the activation of CH_4_ on the Ni-based active sites, and the deposited C* may go to the process of gasification on MgO (Fig. 6). According to the proposed mechanistic pathway, the dissociation, or the activation of CH_4_ as well as the CO_2_, is initiated by the plasma, followed by the elemental and intermediate adsorption composed of C, O, H, and oxy-carbonates on the support surface active sites. The surface-adsorbed species form the desired product in the presence of a catalyst and plasma (Akbari et al. 2017, Messaoudi et al. 2018).

Ray et al. (2018) deployed the DBD plasma reactor with the catalyst to investigate the potential effects on CO_2_–CH_4_ reforming to syngas, and it has been found that incorporating the catalyst into the DBD reactor favored an enhancement in the conversion of CH_4_. The conversion efficiency of CH_4_ was observed to be in the order of Ni-Mn/γ-Al_2_O_3_ > Ni/γ-Al_2_O_3_ > DBD, with the bimetallic catalyst showing the highest CH_4_ conversion probably due to its resistance towards carbon formation. Similar trends were observed for the CO_2_ conversion, i.e., Ni-Mn/γ-Al_2_O_3_ > Ni/γ-Al_2_O_3_ > DBD.

**Fig. 7:**
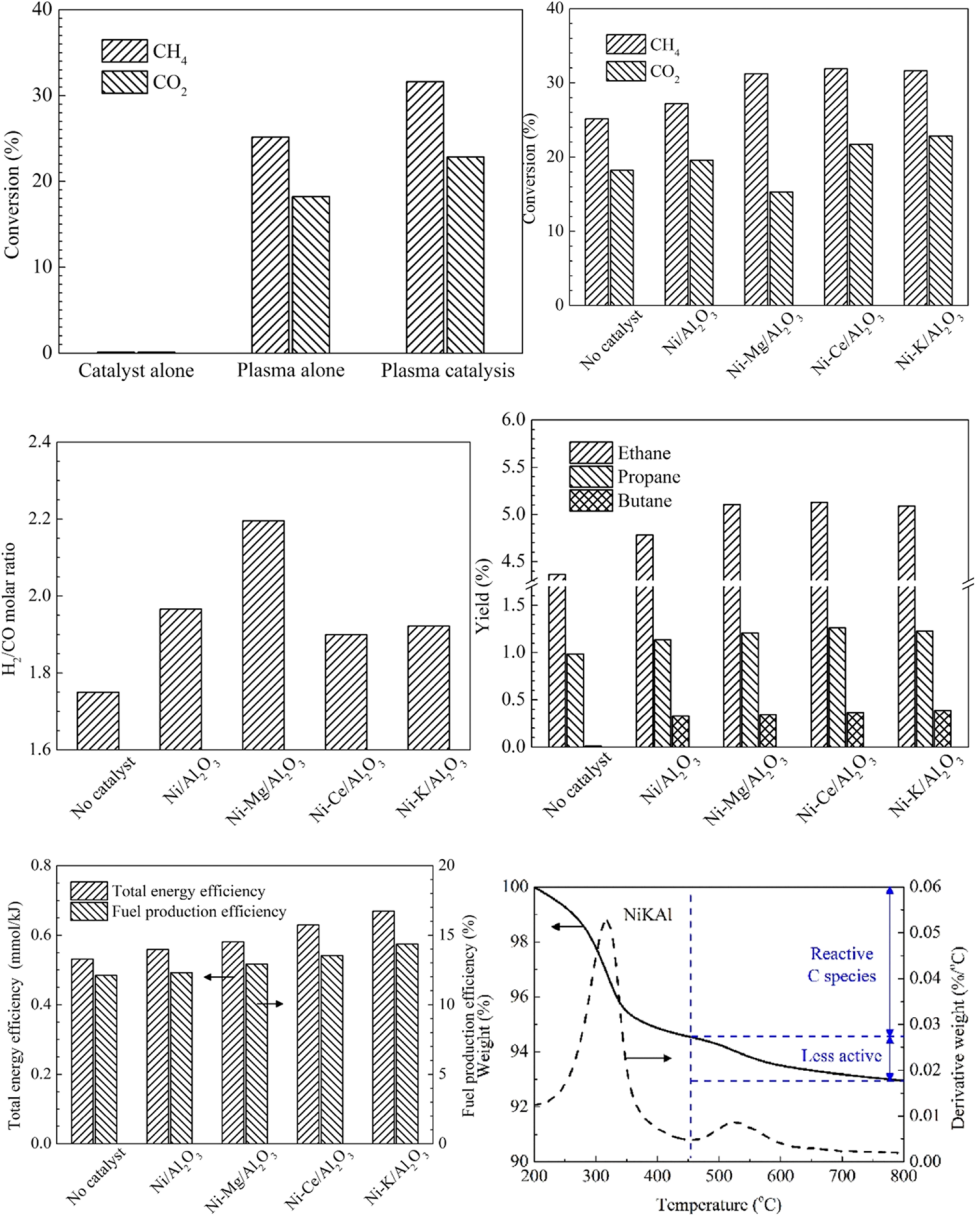
An overview of CO_2_*–*CH_4_ reforming with the effect of plasma and catalysts and promoted catalysts on CO_2_ and CH_4_ conversion, CO, C_2_*–*C_4_ alkanes, and H_2_ yield together with energy efficiency and catalyst stability. Reprinted from the reference Zeng et al. (2018) with the permission of Elsevier

Zeng et al. (2018) investigated CO_2_–CH_4_ reforming to syngas in three different modes: the plasma-only, catalyst-only (Ni/Al_2_O_3_ at 160 °C), and plasma-catalyst mode, to explore the potential effects in a DBD reactor (Fig. 7). The hybrid plasma-catalysis mode demonstrated higher CH_4_ conversion, H_2_ production, and energy efficiency than the plasma-only and catalyst-only (Ni/Al_2_O_3_) modes at 160 °C. Comparing the promoted catalysts with the catalyst (Ni/Al_2_O_3_) showed that the promoted catalysts (Ni/Al_2_O_3_ catalyst modified with K–, Mg–, and Ce–) had higher CH_4_ conversion performance attributed to their enhanced acidic active sites as the acidic active sites are responsible for activating CH_4_. Compared with thermochemical conversion, it has been found that these promoters have adverse effects on CH_4_ conversion. Interestingly, the promoters demonstrated a different behavior in plasma and thermochemical conditions, indicating that their behavior was temperature-dependent regarding carbon deposition and the conversion of CH_4_. Among the promoted catalysts, the Mg-promoted catalyst significantly enhanced the H_2_/CO molar ratio, probably due to the weak CO_2_ affinity to the catalyst’s surface.

In contrast, the K-promoted catalyst demonstrated the overall best performance in CO_2_–CH_4_ conversion and the yields of C_2_–C_4_ alkanes, CO, and H_2_ together with energy efficiency. Promoted catalysts also favored carbon deposition in the hybrid plasma-catalysis process. However, the total quantity of deposited carbon was still lower than in the high-temperature thermochemical reforming of CO_2_/CH_4_. The thermogravimetric analysis (TGA) revealed that the spent catalyst has carbon deposition in the reactive carbon-based species, which can be easily oxidized by O and CO_2_ to maintain the catalyst stability during the reaction.

### CO_2_ dissociation to CO

**Fig. 8:**
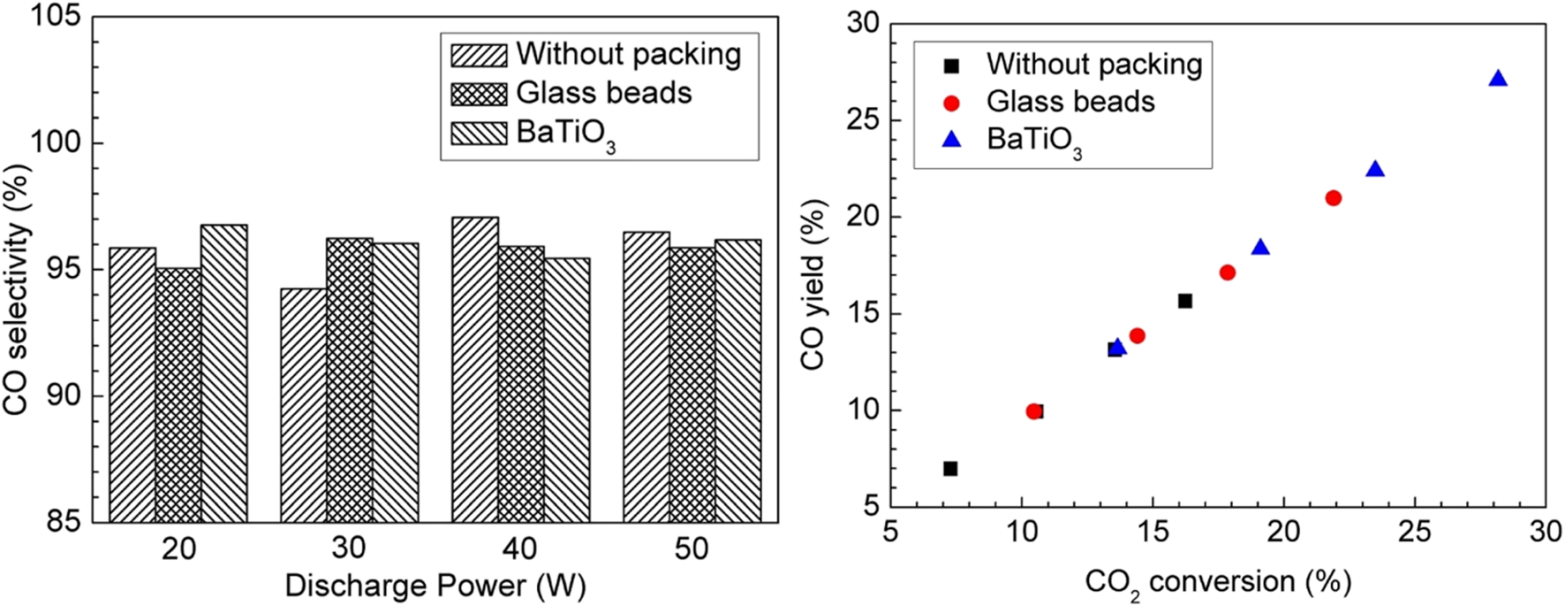
Effect of packing materials on (left) CO selectivity and (right) CO yield. Reprinted from the reference Mei et al. (2014) with the permission of IOP Science Publishing

Mei et al. (2014) reported a plasma catalysis system for CO_2_ conversion into O_2_ and CO in a DBD cylindrical-shaped reactor using and/or without the packing materials. The aim was to investigate the potential effects of the materials used as reactor packing, i.e., glass beads and BaTiO_3_, on the CO_2_ discharge properties and conversion to CO and O_2_. At the same discharge power, it has been found that there was an evolution in the CO_2_ discharge behavior from a typical filamentary discharge with no packing material to a combination of surface discharge with glass beads and barium titanate (BaTiO_3_) packings and filamentary. Incorporating BaTiO_3_ into the plasma system improves the energy of electrons and increases the average electric field by 98.49% and 93.78%, respectively, further influencing the plasma-catalysis–based reactions. In addition, it has been found that utilization of the reactor packing materials, i.e., glass beads and BaTiO_3_, in the discharge gap results in the efficiency enhancement of the DBD reactor for the conversion of CO_2_, although the CO_2_ residence time has been reduced due to an increase in the volume of discharge at the identical flow rate of gas. Overall, it has been found that utilization of the reactor packing materials, i.e., glass beads and BaTiO_3_, significantly improved the conversion of CO_2_ by 75% and the yield of CO due to variation in the discharge properties (Fig. 8), making it an efficient process compared to CO_2_ conversion in plasma catalysis with no packing. Furthermore, the electron with a highly energetic state (> 3.0 eV) produced by the discharge could be assistive in activating the photocatalyst (BaTiO_3_) by an electron-hole pair on to the surface of the catalyst, which further provides a synergistic effect to the CO_2_ conversion process.

In another work, Mei and Tu (2017) extended their research to explore the potential different parametric effects on the plasma-catalysis–based transformation of CO_2_ to O_2_ as well as the CO. These parameters included the discharge length and frequency, plasma power, electrode gap, CO_2_ flow rate, dielectric materials thickness, and the reactor design using a DBD coaxial reactor. It has been found that the decomposition of CO_2_ and the efficiency of the process were not affected noticeably by the discharge frequency. Higher discharge power and/or lower flow rate CO_2_ feed results in an increased conversion of CO_2_ than the lower discharge and/or higher CO_2_ feed flow rate. In this regard, a clear trade-off existed between the conversion of CO_2_ and the plasma process efficiency. In addition, the decomposition of CO_2_ and process energy efficiency was positively affected by lowering the thickness of the dielectric material and discharge gap and increasing the discharge length. Regression modeling shows that the flow rate of the CO_2_ feed and the discharge power are the key drivers for enhancing the conversion of CO_2_ and the process’s energy efficiency.

**Fig. 9:**
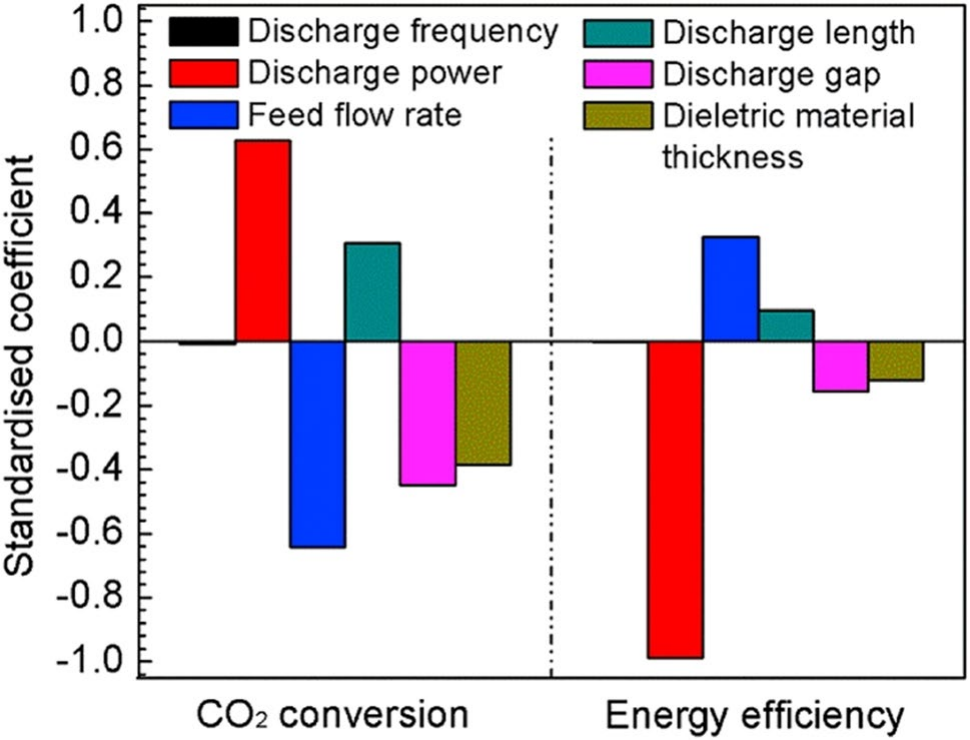
Standardized coefficients for various processing parameters for (left) CO_2_ conversion and (right) energy efficiency. Reprinted from the reference Mei and Tu (2017) with the permission of Elsevier

Additionally, it has been observed that using aluminum (Al) foil on the outer surface of the electrodes and the screw-type inner electrodes composed of stainless steel (SS) significantly improved the conversion of CO_2_ and energy efficiency compared to other forms of electrodes. The role of the SS screw-type inner electrode and Al foil was investigated, and it was found that the Al foil contributed to the enlargement of the effective discharge area. In contrast, the SS screw-type electrodes improved the local electric field near the electrode’s sharp edge. Both effects positively enhanced the process of converting CO_2_ (Fig. 9).

Xu et al. (2017) reported the CO_2_ dissociation in NTP at atmospheric pressure using a reactor packed with BaTiO_3_ packing to investigate the potential impacts of CO_2_ dilution with nitrogen (N_2_) and argon (Ar). The results showed that the packing material composed of BaTiO_3_ ferroelectric in contact with the electrodes facilitated the conversion of CO_2_ with higher yield and the process energy efficiency compared to the DBD reactor with and without packing materials using dielectric layer–covered electrodes. Based on their operating conditions, the CO_2_ dissociation was unaffected and remained constant despite the input energy. Thus, the packing material composed of BaTiO_3_ ferroelectric, together with the NTP atmospheric pressure system for CO_2_ dissociation, was found to be a potential and powerful alternative to CO_2_ utilization compared to thermal catalysis, in terms of energy efficiency, process economics, and scale-up configurations. In addition to the above discussion, the influence of feed CO_2_ dilution with N_2_ and Ar showed a positive impact by increasing CO_2_ conversion by reducing the breakdown voltage at a given energy input for the dissociation of CO_2_, resulting in a simplifying NTP process design in terms of power supply. O_2_ and CO were the significant products identified in the process of dissociation of CO_2_. At the same time, a trace amount of ozone (O_3_) was also detected (maximum 100 ppm) in the case of pure CO_2_ and Ar/CO_2_ systems but was not observed in the case of the N_2_/CO_2_ system. It has been found that the higher dilution of the CO_2_ feed with Ar results in higher production of atomic oxygen that leads to a higher concentration of O_3_, but at the same time, a temperature rise caused by the higher input of the plasma energy results in O_3_ dissociation. However, as mentioned above, there was no O_3_ in the N_2_/CO_2_ system, and nitrogen oxides (N_2_O, NO_2_, and NO) were identified up to a maximum of 3120 ppm. Based on their proposed mechanism, except for higher plasma energies, the N_2_O formation is generally favored compared to NO_*X*_. In the meantime, the higher contents of N_2_ facilitate the NO_2_ conversion to NO and NO conversion to N_2_.

In another similar study (Yap et al. 2015), the effect of CO_2_ dilution using and without the helium (He) was explored experimentally in the plasma catalysis CO_2_ conversion process to CO and O_2_ using glass balls filled with a non-thermal plasma reactor and two different generator supplies. It has been found that the sinusoidal excitation-based discharge generation was much more effective for dissociating CO_2_ than the pulsed generator discharge at an equivalent input power. At specific input energy (40 kJ/mole), 7.5% of CO_2_ conversion was obtained with the pulsed supply while 13.5% with the sinusoidal excitation with a dilution ratio of He/CO_2_ = 1/1. The kinetic investigations revealed the first-order kinetics, but the rate constant significantly differed in the two generator types. From the mechanistic point of view, the CO_2_ dilution with He favors the CO_2_ conversion to CO, particularly in the case of AC sinus activation, which produces mainly species possessing vibrational excitation. However, pure CO_2_ demonstrated remarkable efficiency in both types of power supply. It has been found that the CO selectivity and the carbon balance were maximal with the He dilution. At the same time, there was an issue with the carbon deposition in the case of a pure CO_2_ system without dilution. Large cubic and spherical particles with carbon filaments that were several tens of micrometers long were identified. They have demonstrated for the first time that carbon atoms can also be merged into the silica network of the glass under the NTP discharge during the dissociation of the CO_2_ process, indicating a higher energetic process in plasma. The CO_2_ conversion remains constant, but the CO selectivity improved by controlling the reactor wall temperature, and the lower temperature favors the higher CO selectivity, indicating that the carbon balance is because of the decomposition of CO on the reactor walls.

Butterworth et al. (2016) reported the potential impacts of the particle size of the two distinct packing materials (BaTiO_3_ and Al_2_O_3_) on CO_2_ conversion to O_2_ and CO in the packed bed reactor assisted by plasma catalysis. It has been found that the reduced particle size of the packing materials (180–300 µm) could enhance the conversion of CO_2_ by 70% and also enhance the incidence of reactor partial discharging and reactor breakdown voltage. The term “partial discharging” represents a decline in the reactor fraction where the plasma formation occurs, usually initiated by insufficient applied electric field strength, subsequently reducing the reactor efficacy. Hence, partial discharging can be prevented by providing excessive electric fields in the reactor consuming voltage. For a fair comparative analysis, it should be noted that the comparison of packing materials should be investigated only when the partial discharge is shallow; otherwise, effective materials for reactor packing might be unconsciously neglected. Peeters and van de Sanden developed Lissajous figures based on the partial discharging equivalent circuit that could be used to quantify partial discharging and reactor burning voltage (Peeters and Sanden 2014). In another work (Paulussen et al. 2010), it has been experimentally revealed that the CO_2_ feed flow rate is the utmost critical parameter influencing the conversion of CO_2_ and the yield of CO, which is usually higher at a lower CO_2_ feed flow rate. Additionally, the influence of temperature directly impacts CO_2_ conversion, but CO yield is limited.

### CO_2_ hydrogenation

Wang et al. (2018a) investigated the hydrogenation of CO_2_ over the hybrid catalyst-based NTP by considering the different parametric effects, including reactor design, reactant composition, and catalyst effect. Initially, they developed three different types of DBD reactor arrangements depending on the construction materials for the ground electrode and higher voltage electrode known as reactor-I (aluminum foil with stainless steel (SS) rod), reactor-II (water with SS rod covered by the quartz tube), and reactor-III (water with SS rod). Based on the design of different reactors, it has been found that the overall CO_2_ conversion was quite similar, but the product distribution varied widely. In all reactors, methanol and ethanol were identified as the major oxygenated products, while CO and CH_4_ were the most significant gaseous products. It has been found that reactor III produced the highest methanol compared to reactors I and II, while reactor I resulted in the highest CO selectivity. The significant increase in the methanol production and selectivity using reactor-III could be directed to the water used as a ground electrode instead of aluminum foil, which successfully maintained the temperature. The CO selectivity in reactor-III was the lowest among the three reactors, possibly due to the highest conversion of CO to methanol. As reactor-II and reactor-III operated at lower temperatures, higher activity was observed with oxygenate formation (CH_3_OH). CO was the dominant product in reactor-I due to high-temperature operation. Another advantage of reactor-II and reactor-III at lower temperature operation was that they inhibited the further decomposition of CH_3_OH (Wang et al. 2023b). Reactor III demonstrated the best performance even though high voltage was applied in reactors I and II. The better performance of reactor III was related to the domination of strong filaments, which were much weaker in reactor II. The feed molar ratio with the catalyst was also investigated in their work. It is an admitted fact and observed in their study that the feed molar ratio, i.e., CO_2_:H_2_, significantly influences the methanol conversion, concentration, and yield. By increasing the hydrogen ratio over CO_2_, it has been found that the overall methanol conversion increases with a decrease in the CO selectivity, indicating the forward reaction. In addition to the feed molar ratio, the effect of catalysts was also studied. Two different catalysts, Pt/Al_2_O_3_ and Cu/Al_2_O_3_, were employed for efficient H_2_ and CO_2_ activation, respectively. The results revealed that the Cu/Al_2_O_3_ catalyst demonstrated better performance towards methanol production than the Pt/Al_2_O_3_ catalyst.

It has been known that molecular adsorption depends on the molecule-catalyst interaction, and it is not supposed to be a spontaneous process. Due to the higher internal energy of CO_2_ (v), its adsorption is preferred energetically compared to the CO_2_ in its ground state. However, the plasma characteristics influence the CO_2_ (v) adsorption, including the electron energy and electric field (Wang et al. 2017b). Based on the previously reported studies on CH_4_ conversion using the plasma-catalysis system, the efficient adsorption of CH_4_ (v) species on the Ni metal surface was the origin of the higher CH_4_ conversion compared to the thermochemical process of steam methane reforming. Quantitatively, the CH_4_ conversion was 50% and 20% in the plasma-catalysis and thermochemical conversion processes, respectively (Nozaki et al. 2004). Furthermore, it has been found that the CO_2_ (v) adsorption onto the surface of the catalyst resulted in the dissipation of the CO_2_ (v) energy to the catalyst, leading to the Auger de-excitation and formation of low-energy electron-hole pairs, which could alter the physical and chemical characteristics of the catalyst, including the electronic structure, to make it more active and trigger the CO_2_ (ad) to form HOCO (ad) and HCOO (ad). Additionally, the RWGS can also occur at the surface of the catalyst, preceding the production of CO (ad) from CO_2_ (ad), which could be the possible reason for the enhanced formation of CO when plasma reactors are packed with the catalysts (Porosoff et al. 2016).

Comparing the catalyst performance in the plasma-catalysis mode, it has been found that Cu/γ-Al_2_O_3_ demonstrated better performance in terms of synthesis of methanol (yield) than Pt/γ-Al_2_O_3_ with almost identical OES CO_2_/H_2_ discharge spectra, indicating that the physical and chemical characteristics of the catalysts were more dominant to determine their different reaction performances. It has also been found that the Cu nanoparticle size in Cu/γ-Al_2_O_3_ was considerably more significant than the Pt nanoparticle size in Pt/γ-Al_2_O_3_. However, better performance for methanol synthesis was observed for Cu/γ-Al_2_O_3_ rather than Pt/γ-Al_2_O_3_, indicating that the particle size was not the dominant factor in determining the catalyst performance. At the same time, the x-ray diffraction (XRD) analysis of the spent catalyst demonstrated that the Cu in the metallic state was the dominant phase over the catalyst’s surface, a highly active site for the hydrogenation of CO_2_ to methanol. Compared to the active metal particle size, the oxygen-based intermediates (Δ*E*_*o*_) adsorption energy over the active metal surface was the dominant parameter related to the catalyst performance for the hydrogenation of CO_2_ to CH_3_OH. Based on the theoretical investigation, a volcano chart was developed, which showed that the elements located on the volcano top would favor the hydrogenation of CO_2_ to methanol. Cu is found to be on the top of the volcano, indicating that Cu is the dominant catalyst over Pt and others for CO_2_ hydrogenation to methanol because it will moderately bind the oxygen-based intermediates at atmospheric pressure (Studt et al. 2014). In another study to investigate the CO_2_ hydrogenation mechanism over the Pt nanoparticles supported separately on SiO_2_ and TiO_2_, it has been found that probably due to the weaker CO_2_ binding to the catalyst, the Pt nanoparticles could not catalyze the reaction. However, once the CO_2_ hydrogenation to CO was initiated with the stabilization of CO_2_, the reaction proceeded via RWGR (Kattel et al. 2016). By using an in situ attenuated total reflection infrared (ATR-IR) spectroscopy, it has been found that the CO_2_ adsorption leads to the synthesis of carbonate-like species over the Pt/γ-Al_2_O_3_ and γ-Al_2_O_3_, which are subsequently hydrogenated to produce CO as a final product (Ferri et al. 2002). The abovementioned findings indicated why the Pt/γ-Al_2_O_3_ resulted in higher CO selectivity than the Cu/γ-Al_2_O_3_ or plasma-only mode.

### CO_2_ to organic acids

Electrochemical reduction of CO_2_ (ITo et al. 1982) to produce carboxylic acids was investigated a long time ago by using the non-aqueous electrolytes composed of dimethyl sulfoxide (DMSO) as an aprotic solvent with tetraalkylammonium salts over different electrodes such as lead (Pd), indium (In), zinc (Zn), and strontium (Sn). Based on the experimental investigation, it has been found that oxalic acid was the dominant product of the CO_2_ electrochemical reduction with Pd electrodes. However, other higher carboxylic acids, including the propionic, glycolic, malonic, and *n*-butyric acids, were produced in addition to the formic acid at substantial concentrations in TEAP or TEABr/DMSO because oxalic, tartaric, and formic acids were found to be the dominant products in the case of TBABr/DMSO, with a negligible amount of other organic acids produced. CO was identified as a dominant product with a low oxalic, formic, malonic, and glycolic acid concentration in the Zn, Sn, and In electrodes. Based on experimental evidence, it has been confirmed that oxalic acid as an intermediate pathway can be used to produce the higher carboxylic acids mentioned above through CO_2_ cathodic reduction.

Recently, the electrochemical CO_2_ reduction to produce formate and oxalate facilitated by the solvated electrons produced by the plasma operated at atmospheric pressure was examined (Rumbach et al. 2016). In this work, the optical absorbance measurements were employed to visualize the solvation of free electrons produced by the plasma at atmospheric pressure into the solution to reduce the aqueous CO_2_ to carboxyl radical anion, i.e., CO_2_^−^ (aq.). It was found that most of the CO_2_^−^ (aq.) ions were typically recombined to produce oxalate under basic conditions. In contrast, some of them produced formate following the disproportionation mechanism with the kinetics of this reduction reaction, such as bulk kinetics in radiolysis experimentations. The formate was found to be the dominant species under strongly acidic conditions. Higher concentrations of dissolved CO_2_ could help improve the process efficiency, but there will be a trade-off between the oxalate and formate yields due to pH. In another similar work (Ihara et al. 1994), the reverse combustion, i.e., the reduction of CO_2_ in water assisted by plasma, was investigated, and the oxalic acid with hydrogen peroxide (H_2_O_2_) was identified as a significant product with low yield with no formation of alcohol or mixture of acids.

### One-step CO_2_–CH_4_ reforming to liquid chemicals

Wang et al. (2017c) investigated the one-step CO_2_–CH_4_ reforming into a series of liquids (CH_3_COOH, CH_3_OH, C_2_H_5_OH, and C_3_H_6_O), with CH_3_COOH as the dominant product. A trace amount of HCOOH, propanol (C_3_H_8_O), and butanol (C_4_H_9_OH) were also identified. The gaseous products included H_2_, CO, and C_*x*_H_*y*_ (where *x* = 2–4 and *y* = 2–10). In this reaction, it was found that the catalyst could not trigger the reaction at 30 °C. At the same time, the NTP assisted the unfavorable reaction, i.e., thermodynamics, in proceeding at ambient temperature. It was observed that combining the catalyst with the plasma could help employ the synthesis and distribution of various oxygenates under ambient reaction conditions. CH_3_COOH was the dominant product, and whatever catalyst was employed for this reaction was followed by CH_3_OH and C_2_H_5_OH. HCOOH was formed only in the case of catalysts supported by noble metals with the highest selectivity over the Pt/γ-Al_2_O_3_. The distribution of the gaseous product was not influenced by adding the catalyst into the plasma-only mode, with CO, H_2_, and C_2_H_6_ being dominant gaseous products. In hybrid plasma catalysis mode, the H_2_ selectivity was found to be enhanced with slightly increased productivity of C_2_H_6_ with a weak influence on the enhancement of selectivity for CO and other C_*x*_H_*y*_ (where, *x* = 2–4 and *y* = 2–10) except for the catalyst (Cu/γ-Al_2_O_3_). The CO_2_ and CH_4_ conversion decreased slightly in the hybrid plasma catalysis mode compared to the plasma-only mode. It could be due to the variation in the discharge behavior prompted by the catalyst that has an adverse impact on the reaction. Remarkably, a chemical, C_6_H_12_O_4_ (CAS No. 49653-17-0), was identified on the inner walls of the reactor in the hybrid plasma catalysis mode. The proposed experimental setup directly reformed CO_2_–CH_4_ into a series of gaseous and liquid chemicals at ambient reaction conditions, bypassing the syngas formation.

**Fig. 10:**
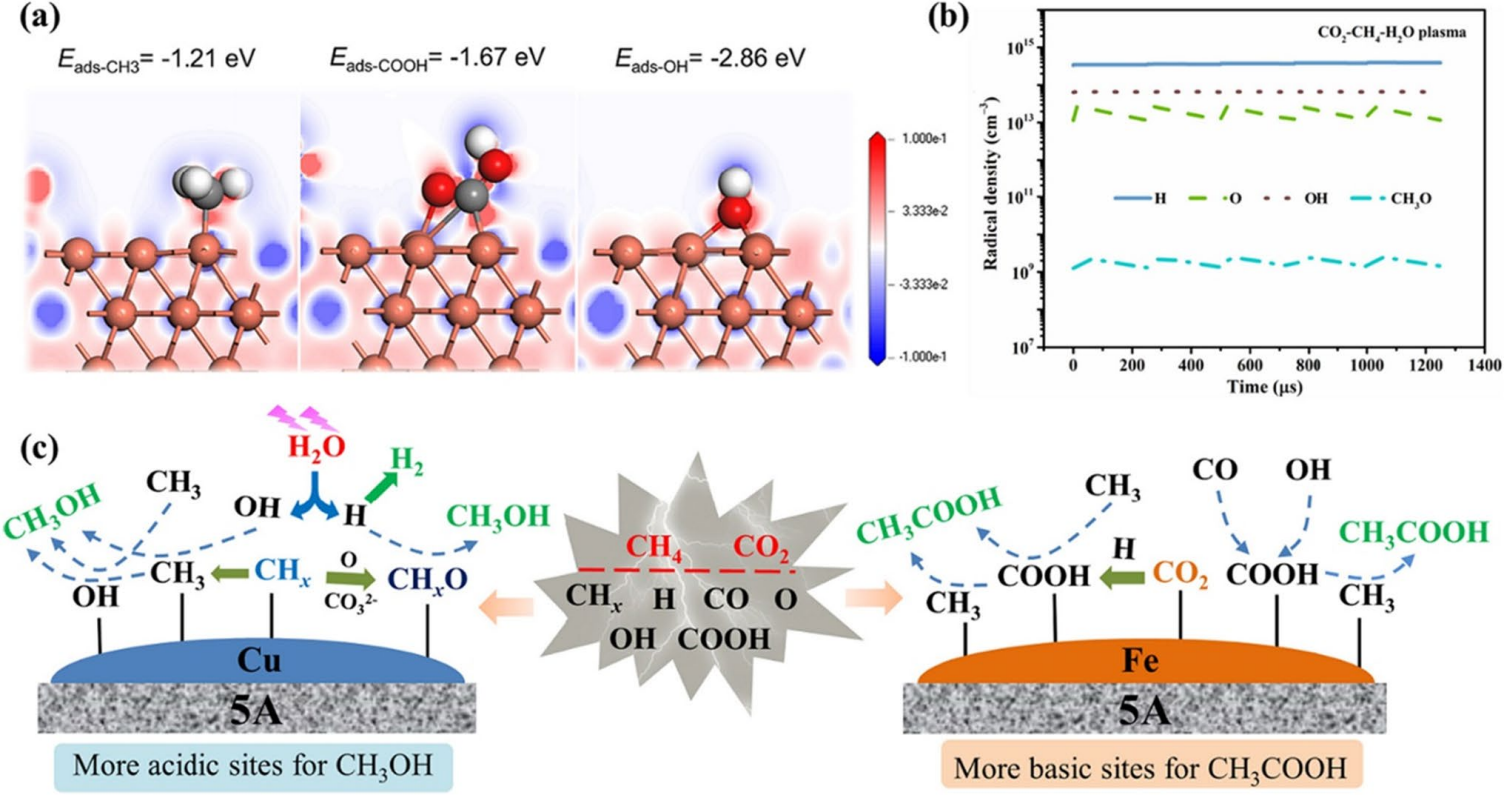
**a** Energy density difference for the CH_3_, COOH, OH radicals with Cu (111) surface; **b** essential radicals’ density as a function of the time during the five periods for the CO_2_–CH_4_–H_2_O plasma; and **c** the discriminative formation of CH_3_OH and CH_3_COOH over Cu/5A and Fe/5A, respectively, via a synergistic plasma-catalyst effects. Reprinted from the reference Li et al. (2023) with the permission of Elsevier

Li et al. (2023) reported an experimental study to synthesize H_2_ and liquid fuels using hybrid plasma catalysis-driven one-step CO_2_–CH_4_ reforming using a Fe- and Cu-based active site catalyst supported on 5A zeolite. The process resulted in a one-stage CO_2_–CH_4_ mixture co-conversion into liquid chemicals and H_2_ due to the strong cooperative effect of the Fe/Cu active sites with the externally applied plasma field. In the case of gaseous products such as CO and H_2_, the selectivity was found to be enhanced (36 to 56.6% for H_2_ and 50 to 57.6% for CO) with the addition of H_2_O, which could be related to the improved radical density of OH and H. In the case of liquid fuels such as CH_3_OH and acetic acid (CH_3_COOH), a strong cooperative effect between the plasma and the acid/base characteristics of the employed catalyst was observed with varied selectivity of the liquid chemicals. A combined in situ diffuse reflectance infrared Fourier transform spectroscopy (DRIFTS) with OES was used to investigate the role of active sites and the possible mechanistic pathways. It was found that the Cu/5A zeolite with a higher density of acidic sites significantly facilitated the production of different carbonate species, which further played their critical role in the synthesis of CH_*x*_O and further CH_3_OH by the recombination of CH_*x*_O with gaseous H_2_. It was found that the COOH generated from both processes, i.e., recombination of gaseous OH with CO and CO_2_ protonation, was the key to producing CH_3_COOH. It was interesting to know that the Cu/5A zeolites (Cu^0^) with a higher acidic sites density and the Fe/5A zeolites (Fe^2+^) with a higher density of basic sites favored the formation of CH_3_OH and CH_3_COOH, respectively (Fig. 10). In addition to the experimental demonstration, the adsorption energy differences–based density functional theory (DFT) calculations for the O-containing radicals showed crucial interactions with the varied charge transfer between the active sites, i.e., Cu (111) surfaces and the gaseous radicals, thus leading to the formation of targeted products. As discussed in this review, a detailed comparison of the plasma- and plasma-catalysis–based CO_2_ conversion routes to C1 products is tabulated in Table 3 with experimental setup and reaction conditions.

**Table 3:** Summary of experimental setup and reaction conditions for CO_2_ conversion to different projects

Discharge type	Catalyst	Feed gases	Feed composition	Major products	CO_2_ conversion	Selectivity	Ref.
MR^a^	N/A	CO_2_/H_2_O	50:50	CO, H_2_	N/A	N/A	Chen et al. (2015)
PCD^b^	NiCr	CO_2_/H_2_O	N/A	CH_4_	76	99	Hoeben et al. (2015)
GD^c^	N/A	CH_4_/CO_2_	9:1	CO, C_2_H_6_, C_2_H_4_, C_3_H_8_, H_2_	52.8	27.8^d^ (47.9^e^)	Huang et al. (2000)
DBD^f^	N/A	CH_4_/CO_2_	34:66	CO, H_2_, C_2_-C_5_, C_6_-C_11+_, oxygenates	N/A	0.2^g^	Li et al. (2002)
RF^h^	N/A	CH_4_/H_2_	1:2	H_2_, CO_2_, CH_4_, CO	46.4	56.9^i^	Patiño et al. (2005b)
AGP^j^	N/A	CH_4_/CO_2_	50:50	CO, H_2_	83.2	80^d^ (90^e^)	Qi et al. (2006)
DBD^f^	Perovskite LaNiO_3_	CH_4_/CO_2_	60:40	CO, H_2_, C_2_H_6_, C_2_H_4_, C_2_H_2_, C_3_H_8_, C_4_-C_6_, oxygenates	13.3	40.5^d^	Goujard et al. (2009)
GAD^k^	Al	CH_4_/CO_2_	50:50	CO, H_2_	8.4	69.5^d^ (31.4^e^)	Tu and Whitehead (2014)
DBD^f^	Ni/γ-Al_2_O_3_	CH_4_/CO_2_	1:9	CO, H_2_, C_2_H_6_	42^l^	37^4^ (33^5^)	Zeng et al. (2015)
DBD^f^	N/A	CH_4_/CO_2_	10:90	CO, H_2_	84	N/A	Snoeckx et al. (2015)
CD^m^	N/A	CH_4_/CO_2_	4/1	CO, H_2_	32	70^d^ (35^e^)	Nguyen et al. (2015)
DBD^f^	TiO_2_/g-C_3_N_4_	CH_4_/CO_2_	1:6	CO, H_2_	20	50^d^ (40^e^)	Lu et al. (2017)
PD^14^	10% La_2_O_3_/Alumina Balls	CH_4_/CO_2_	3.3/6.7	CO, H_2_, Hydrocarbons	11.8	72.1^d^ (24.6^e^)	Yap et al. (2018)
DBD^f^	Ni/γ-Al_2_O_3_-MgO	CH_4_/CO_2_	1:1	CO, H_2_	73	30.5^d^ (29.5^e^)	Khoja et al. (2018)
DBD^f^	Ni-Mn/γ-Al_2_O_3_	CH_4_/CO_2_	1:1	CO, H_2_	13.2	40.5^d^ (13.2^e^)	Ray et al. (2018)
DBD^f^	Ni-K/Al_2_O_3_	CH_4_/CO_2_	3:2	CO, H_2_, C_2_H_6_, C_3_H_8_	22.8	43.3^d^ (31.3^e^)	Zeng et al. (2018)
DBD^f^	BaTiO_3_	CO_2_	Pure	CO, O_2_	75	97^d^	Mei et al. (2014)
DBD^f^	N/A	CO_2_	Pure	CO, O_2_	27.2	94^d^	Mei and Tu (2017)
DBD^f^	BaTiO_3_	CO_2_/Ar	20:80	CO, O_2_, O_3_	36	N/A	Xu et al. (2017)
DBD^f^	N/A	CO_2_	Pure	CO, O_2_	5.4	58^d^	Yap et al. (2015)
DBD^f^	Cu/γ-Al_2_O_3_	CO_2_/H_2_	1:3	CH_3_OH	11.3^15^	53.7^p^	Wang et al. (2018a)
DBD^f^	γ-Al_2_O_3_	CO_2_/CH_4_	1:1	CH_3_COOH, liquid chemicals	15.4	50-60^q^	Wang et al. (2017c)
DBD^f^	Cu-Fe/5A zeolite	CO_2_/CH_4_	1:1	H_2_, CH_3_COOH, CH_3_OH	30.3	56.6^e^	Li et al. (2023)

^a^Microwave radiation

^b^Pulsed corona discharge

^c^Glow discharge

^d^CO selectivity

^e^H_2_ selectivity

^f^Dielectric barrier discharge

^g^C_2_H_4_ selectivity

^h^Radio frequency

^i^C_2_ product selectivity

^j^Abnormal glow plasma

^k^Gliding arc discharge

^l^CH_4_ conversion

^m^Corona discharge

^n^Plasma discharge

^o^CH_3_OH yield

^p^CH_3_OH selectivity

^q^Total selectivity of liquid chemicals

## Conclusions and future recommendations

This review comprehensively analyzed and compared the fundamentals of plasma technology for converting CO_2_ to fuel-graded and value-added products with other conventional CO_2_ conversion technologies. A detailed discussion was conducted on the different types of plasma, and their comparisons based on their characteristics, operational conditions, and performance were also reviewed, as well as various configurations of the plasma reactors. Critical discussions on plasma catalysis for CO_2_ conversion were presented. Some challenges in this field are identified and provided below:

- CO_2_ circular economy: CO_2_ is essential for life on Earth; however, the exponential growth of gases after industrialization has resulted in excessive CO_2_ in the Earth’s atmosphere. The circular carbon economy is a crucial concept for addressing the issue of excessive CO_2_ emissions worldwide. A circular carbon economy is a conceptual structure to regulate and minimize emissions. The closed-loop system incorporates the 4Rs: reduce, reuse, recycle, and remove.
- CO_2_ reduction in H_2_O: It is essential to investigate the reaction mechanism and the process parameters that lead to the H_2_O dissociation in the plasma and the plasma-catalysis systems. Further studies are recommended to determine the vibrational and electron temperatures. For economic feasibility, it is essential to perform real-time optimization of the discharge parameters, including pulse frequency and duration, as it is believed that optimizing these parameters will positively affect energy efficiency. Adding H_2_O weakens the discharge, so experimental studies are required to investigate the Ar effects in the gas mixture, which could be beneficial to sustain the discharge. To further improve conversion and the energy efficiency of CO_2_ reduction in the H_2_O process, the impact of catalysts in plasma should be thoroughly investigated.
- CH_4_ reforming with CO_2_ to syngas: It has been found that the selection of an efficient catalyst to get synergy with the plasma for higher conversion and energy efficiency is still inadequate, and more research is projected to overcome the conventional barriers and make a bridge between the thermal- and plasma-catalysis. It has been established that the metal-supported catalysts are promising candidates in plasma catalysis for the efficient CH_4_ reforming with CO_2_ to syngas, and there is room to develop multifunctional metal-supported catalysts such as bimetallic catalysts of monometallic catalysts in combination with some promoters. Such advanced and next-generation catalysts should be developed to achieve multiple targets simultaneously, including energy efficiency, enhanced conversion, selectivity, and a balanced CO/H_2_ ratio. Investigations on optimization of the discharge effects, input power, and feed flow conditions should be done as they could directly impact the syngas formation, electron density distribution, and conversion/selectivity. Research should be carried out comprehensively to inhibit the carbon deposition observed with higher feed concentrations of CH_4_ by introducing some oxygen to the system. In terms of energy efficiency, there is a need to find a way to inhibit water formation by understanding the mechanisms of water formation.
- CO_2_ dissociation to CO: It has been suggested that the reaction parameters should be optimized to enhance CO_2_ dissociation in plasma catalysis. These parameters include but are not limited to the dielectric materials, the reactor’s geometrical configurations, the catalyst bed, and input power. To date, it has been demonstrated that using a DBD reactor, the CO_2_ dissociation is reasonable; however, the energy efficiency is still too low and needs significant improvement for commercialization if the electricity is produced from fossil fuels. However, having electricity from renewable sources might address the energy efficiency issue. Specifically, the effects of the co-reagents, such as CH_4_ or H_2_O, which are highly probable for the selective formation of value-added chemicals from CO_2_ dissociation, should be investigated using plasma catalysis.
- CO_2_ hydrogenation: It is evident that the hydrogenation of CO_2_ to methanol in the plasma-catalysis system depends on a wide range of reactive species, which could initiate more reaction routes for methanol formation. A detailed investigation should be carried out to govern the interaction between the active sites on the catalyst and the reaction intermediates. The final product selectivity cannot be determined without first determining the adsorption energies of the oxygen-based reaction intermediates to the catalyst surface.
- CO_2_ to organic acids: More comprehensive research is necessary to deliberate the possible reaction mechanistic pathways of CO_2_ conversion to organic acids, such as formic, acetic, and oxalic acid, in connection with the plasma-catalysis synergistic effects. In this regard, developing next-generation multifunctional catalysts for CO_2_ reduction and the coupling of C–C simultaneously is required. The impact of either with or without base use should be investigated regarding product distribution and reaction efficiency.
- One-step CO_2_–CH_4_ reforming to liquid chemicals: Considering the C1 products, such as methanol, the bond energies of C–O (326 kJ/mole) and C–H (416 kJ/mole) in methanol and CH_4_, respectively, suggest that the methanol is less stable in plasma than CH_4_, thus pointing to the need for more fundamental research regarding the potential role of CH_2_O and methanol as reaction intermediates in plasma-catalysis. Developing next-generation selective and efficient catalysts that could produce multi-carbon products from CO_2_ conversion in plasma catalysis is necessary. Thus, parallel process optimization is required for energy efficiency.

## Data Availability

Not applicable.
